# Sufentanil: a risk factor for lactic acidosis in patients after heart valve surgery

**DOI:** 10.1186/s13019-022-01986-5

**Published:** 2022-09-09

**Authors:** Yu-Fei Zhan, Quan Shi, Yu-Chen Pan, Bao-Shi Zheng, Yi-Peng Ge, Tian-Ge Luo, Zhi-Hong Xiao, Wei Jiang

**Affiliations:** 1 Department of Cardiothoracic Surgery, The Second Affiliated Hospital of Guilin Medical University, Guilin, 541199 People’s Republic of China; 2 Anesthesiology Department, First Affiliated Hospital of Guilin Medical University, Guilin, 541001 People’s Republic of China; 3 Department of Cardiac Surgery, Anzhen Hospital, Capital Medical University, Beijing, 100029 People’s Republic of China; 4 Cardic Centre, 924 Hospital of the Chinese Joint Service Support Force, Guangxi Institute of Metabolic Diseases, Guilin, 541002 People’s Republic of China; 5 Department of Cardiothoracic Surgery, First Affiliated Hospital of Guangxi Medical University, Nanning, 530213 People’s Republic of China; 6 Department of Anatomy and Histology, School of Basic Medical Science, Shenzhen University Health Science Centre, Shenzhen, 518055 People’s Republic of China

**Keywords:** Lactic acidosis, Cardiopulmonary bypass, Heart valve surgery, Sufentanil

## Abstract

**Backgrounds:**

Hyperlactatemia is a common metabolic disorder after cardiac surgery with cardiopulmonary bypass. Epinephrine use has been identified as a potential cause of increased lactate levels after cardiac surgery. Stress can lead to an increase in catecholamines, mainly epinephrine, in the body. Exogenous epinephrine causes hyperlactatemia, whereas endogenous epinephrine released by stress may have the same effect. Opioids are the most effective anesthetics to suppress the stress response in the body. The authors sought to provide evidence through a retrospective data analysis that helps investigate the relationship between intraoperative opioid dosage and postoperative lactic acidosis after cardiac surgery.

**Methods:**

The clinical data of 215 patients who underwent valvular heart surgery with cardiopulmonary bypass from July 2016 to July 2019 were analyzed retrospectively. Blood lactate levels were measured at 0.1 h, 2 h, 4 h, and 8 h after surgery. Patients with continuous increases in lactate levels and lactate levels exceeding 5 mmol/L at two or more time points were included in the lactic acidosis group, whereas the other patients were included in the control group. First, univariate correlation analysis was used to identify parameters that were significantly different between the two groups, and then multivariate regression analysis was conducted to elucidate the independent risk factors for lactic acidosis. Fifty-one pairs of patients were screened by propensity score matching analysis (PSM). Then, lactic acid levels at four time points in both groups were analyzed by repeated measures ANOVA.

**Results:**

he EF (heart ejection fraction) (OR = 0.94, *P* = 0.003), aortic occlusion time (OR = 10.17, *P* < 0.001) and relative infusion rate (OR = 2.23, *P* = 0.01) of sufentanil was an independent risk factor for lactic acidosis after valvular heart surgery. The patients were further divided into two groups with the mean sufentanil infusion rate as the reference point. The data were filtered with PSM (Propensity Score Matching). Lactic acid values in both groups peaked at 4 h after surgery and then declined. The rate of lactic acid decline was significantly faster in the group with a higher sufentanil dosage than in the lower group. The difference was statistically significant (*P* < 0.05). There was also a significant difference in lactic acid levels at the four time points (0.1 h, 2 h, 4 h and 8 h after surgery) in both groups (*P* < 0.001).

**Conclusion:**

The inadequate intraoperative infusion rate of sufentanil is an independent risk factor for lactic acidosis after heart valve surgery. The possibility of lactic acidosis caused by this factor after cardiac surgery should be considered, which is helpful for postoperative patient management.

## Introduction

The main cause of death in the early postoperative period in patients undergoing heart valve surgery is multiple organ dysfunction syndrome (MODS) [1]. The pathophysiological basis for postoperative MODS is cellular damage, which is manifested when cellular repair does not occur. During operations the oxygen consumption is inadequate to meet intraoperative metabolic requirements [1]. Hypoxia may result from insufficient blood supply caused by decreased cardiac output, reduced hemoglobin levels, or impaired absorption of oxygen by target cells. Determination of lactate blood level is helpful in diagnosis and assessment of hypoxia and lactic acidosis in people in shock or heart failure [1]. Hyperlactatemia is a common metabolic disorder after cardiac surgery with cardiopulmonary bypass [2–5]. Its mechanism is complex and controversial [6]. Tissue hypoxia is the most common cause of hyperlactatemia (type A). However, studies have shown that type B (absence of tissue hypoxia) may also be a cause of hyperlactatemia after heart surgery [7]. In particular, some studies have shown that epinephrine can cause hyperlactatemia, which is called epinephrine-induced hyperlactatemia [2, 7–10]. Its mechanism is called aerobic glycolysis. The cause of this glycolysis is not a lack of oxygen but a response to stress. It can produce ATP quickly and efficiently [11]. Heart surgery trauma can lead to severe stress reactions in the body. Opioids are the most effective anesthetics to suppress the stress response in the body. Sufentanil is a commonly used opioid analgesic in the clinic, especially in our center. Therefore, the purpose of this study was to determine the relationship between intraoperative sufentanil dosage and postoperative lactic acidosis.

## Materials and methods

### Data collection

Data were collected retrospectively from all patients primarily undergoing heart valve surgery at the authors' institution (The 924 Hospital of the Chinese Joint Service Support Force) from July 2016 to July 2019. A total of 215 patients (115 males and 100 females; mean age 54.47 ± 12.95 years [range, 15–79 years]) with valvular heart diseases were enrolled. The primary diseases and surgical status of the patients are shown in Table 1. All operations were performed with median sternotomy and cardiopulmonary bypass. The following preoperative factors were examined: sex, age, body surface area, EF, and left ventricular end-diastolic diameter. The following intraoperative factors were analyzed: cardiopulmonary bypass time, aortic occlusion time, total dosage of propofol, total dosage and relative infusion rate of sufentanil, and total operative time. Blood lactate levels were measured 0.1 h, 2 h, 4 h and 8 h after surgery using a Radiometer blood gas analyzer (Brønshøj, Denmark). Hyperlactatemia was defined as lactic acid > 3 mmol/L, and lactic acid greater than 5 mmol/L was defined as lactic acidosis. Considering that continuously increased lactate levels are a better indicator of metabolism than a single value, patients who exhibited continually elevated lactate levels and lactate levels exceeding 5 mmol/L at two or more time points were included in the lactic acidosis group in this study, and the remaining patients were included in the control group.

**Table 1:** Patients, Preoperative and Inoperative Characteristics and Descriptive Data and results of univariate analysis and chi-square test

Variable	Lactic acidosis group (n = 38)	Control group (n = 177)	*P*
Female	21 (55.2)	79 (44.6)	0.28
Age (years)	57.65 ± 9.27	53.79 ± 13.53	0.1
Body surface area (m^2^)	1.72 ± 0.1	1.72 ± 0.22	0.85
Main preoperative diagnosis (case)			
RHD	33	133	
DHVD	3	42	
IE	2	2	
Preoperative diabetes mellitus	3	24	0.26
Preoperative atrial ibirllation fibrillation	12	40	0.17
Preoperative hypertension	10	45	0.53
Operative method			
MVP + TVP	6	33	
MVP	0	5	
MVP + AVR	0	1	
MVR + TVP	24	123	
AVR	2	13	
MVR + AVR + TVP	6	2	
Preoperative EF value (%)	60 (50.75, 62.75)	62 (60, 67)	0.01
Preoperative left ventricle (end-diastolic diameter cm)	54 (50.75, 56)	51 (48, 55)	0.02
Cardiopulmonary bypass time (min)	140.05 ± 60.05	117.05 ± 57.45	< 0.001
Aortic occlusion time (min)	104 ± 46.76	84.20 ± 43.83	< 0.001
Total operation time (min)	240 (180, 300)	240 (205, 300)	0.79
Total sufentanil dosage (µg)	545.26 ± 163.05	478.13 ± 146.51	0.01
Relative sufentanil infusion rate (µg.kg-1.h-1)	2.15 ± 0.8	1.83 ± 0.60	0.03
8 h mean blood glucose level(mmol/L)	15.37 ± 2.87	12.79 ± 2.01	0.19
Infusion rate of propofol(mg/kg.h)	7 (6.6, 8.4)	6.9 (6.5, 8)	0.95
8 h Total dopamine dosage(mg)	166.8 (118.4, 174.6)	158.4 (129.6, 184.3)	0.89
8 h Total Epinephrine dosage(mg)	7 (18.4)	38 (21.4)	0.67

Data are presented as numbers, the mean ± standard deviation or median (interquartile range). Abbreviations: RHD, rheumatic heart disease; DHVD, degenerative heart valvular disease; IE, infective endocarditis; MVP, mitral valvuloplasty; MVR, mitral valve replacement; TVP, tricuspid valve replacement; AVR, aortic valve replacement. Among the patients, 52 also presented with atrial fibrillation before surgery, 45 of whom underwent radiofrequency ablation (Maze IV)

### Clinical management

All surgeries were performed via median sternotomy. Anesthesia techniques and medications were similar. Mild hypothermia (32 °C) was induced during cardiopulmonary bypass, and HTK solution was used to ensure myocardial protection during the operation. The intraoperative cardiopulmonary bypass perfusion pressure was 50–80 mmHg. Patient temperature was normalized (nasal temperature, 37 °C; rectal temperature, 36 °C) after cardiopulmonary bypass. General anesthesia was total intravenous anesthesia, and the volatile anesthetic agent was only temporarily used for blood pressure control. Sufentanil was used for anesthesia induction and maintenance. The induction dose was 1 µg/kg, and then intraoperative infusion continued until the end of the operation. The infusion rate was adjusted as required. The total sufentanil dosage and the relative infusion rate of sufentanil ([total sufentanil/operative time (min)/body weight] × 60) were calculated. The continuous infusion of propofol was used for sedation. The infusion rate of propofol was 4–10 mg kg^−1^ h, which was adjusted according to BIS. All operations went well. Dopamine was routinely used after the heart beat recovered, and its infusion rate was 3–8 µg kg^−1^.min. Epinephrine was used for patients whose systolic pressure was less than 85 mmHg with dopamine and enough liquid volume. Its infusion rate was 0.01–0.08 µg kg^−1^ min. Propofol was used for sedation in patients with postoperative agitation. None of the patients received opioid analgesics within 8 h after surgery. Continuous infusion of insulin was routinely used to control blood glucose.

### Statistical methods

SPSS ver. 25.0 statistical software was used for analysis. Descriptive data are expressed as the mean ± standard deviation, proportions, or median (interquartile range). For univariate analysis, an independent-samples t-test was used to analyze the normally distributed continuous data, and the nonparametric Mann-Whitey U test was used for nonnormally distributed continuous data. Normal distribution was determined using the Kolmogorov–Smirnov test. The CPB time and aortic occlusion time data were divided into two groups according to the cutoff of the 95% percentile. Then, the chi-square test was performed on the two groups of data, and the lactic data, risk factors identified via univariate analysis and the chi-square test, were included in the multivariate regression model. Then, logistic multivariate stepwise regression analysis was conducted to identify the independent factors associated with lactic acidosis after cardiopulmonary bypass.

The authors identified that the inadequate intraoperative infusion rate of sufentanil was an independent risk factor for lactic acidosis in heart valve surgery patients. The relative infusion rate of sufentanil was normally distributed, and the patients were further divided into two groups with the mean sufentanil infusion rate (1.88 µg kg^−1^ h) as the cutoff point. Fifty-one pairs of patients were screened by propensity score matching analysis (PSM). Then, lactic acid levels at four time points (0.1 h, 2 h, 4 h and 8 h after surgery) in both groups were analyzed by repeated measures ANOVA. A *P* value ≤ 0.05 was considered to be statistically significant.

## Results

Among the 215 patients, 38 patients (17.6%) exhibited continuously increased lactate levels after surgery as well as lactate levels exceeding 5 mmol/L at two or more time points. The majority of patients (62.5%) had elevated blood lactate > 2 mmol/L on ICU admission. Within 24 h, the lactate values of 95.3% of patients returned to normal levels. The mortality was low (0.9%).

### Risk factors associated with lactic acidosis

Univariate analysis and chi-square test illustrated that increased lactate levels were significantly correlated with EF (*P* = 0.01), preoperative left ventricle (end-diastolic diameter) (*P* = 0.01), aortic occlusion time (*P* < 0.001), cardiopulmonary bypass (CPB) time (*P* < 0.001), sufentanil dosage (*P* = 0.01) and the sufentanil relative infusion rate (*P* = 0.03) (Table 1).

After univariate correlation analysis, the statistically significant factors were included in the multivariate regression model. Logistic multivariate stepwise regression analysis revealed that EF (*P* = 0.003, OR = 0.94), aortic occlusion time (*P* < 0.001, OR = 10.17), and sufentanil relative infusion rate (*P* = 0.01, OR = 2.23) were independent risk factors associated with lactate acidosis (Table 2).

**Table 2:** Logistic regression analysis of factors associated with lactic acidosis after valvular heart surgery involving cardiopulmonary bypass

Variable	OR	95% CI	P value
EF (%)	0.94	0.89–0.97	0.003
Aortic occlusion time (95%) (min)	10.17	3.86–26.75	< 0.001
Relative sufentanil infusion rate (µg kg^−1^ h^−1^)	2.23	1.26–3.93	0.01

Input variables: EF, Preoperative left ventricle (end-diastolic diameter cm), Aortic occlusion time (95%) (min), Cardiopulmonary bypass time (95%) (min), Total sufentanil dosage (µg) and sufentanil relative infusion rate (µg kg^−1^ h^−1^)

Sufentanil relative infusion rate = (total sufentanil/operative time [min]/body weight) × 60

### The relationship between the sufentanil relative infusion rate and postoperative lactic acidosis

**Fig. 1:**
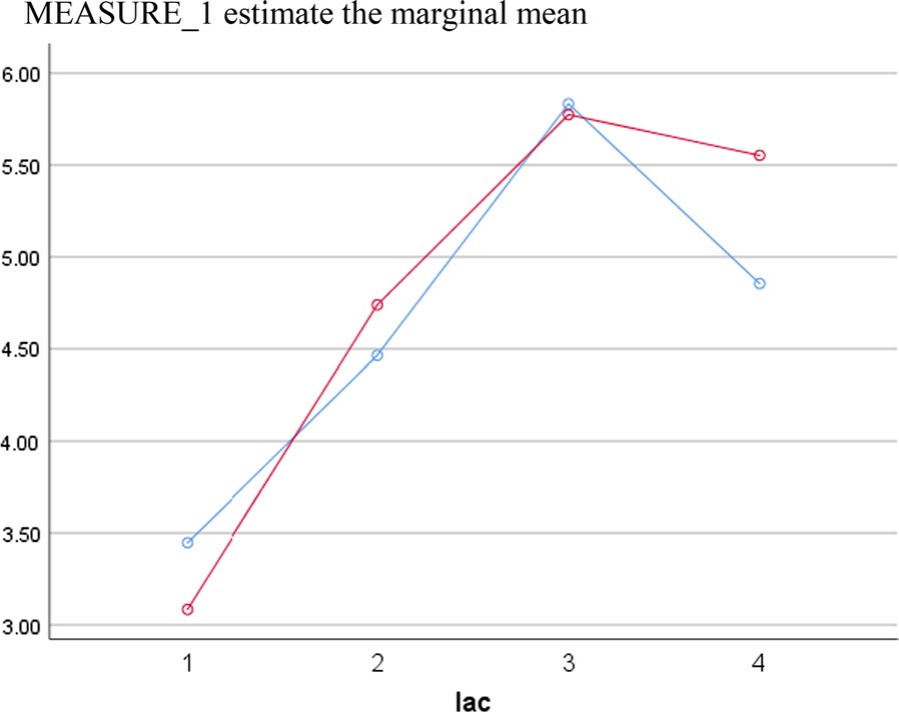
Lactic acid comparison between two groups with different infusion speeds of sufentanil (Red line: The infusion rate of sufentanil is less than 1.88 µg/kg h; Blue line: The infusion rate of sufentanil is greater than 1.88 µg/kg h). Blood lactate levels were measured at 10 min and 2 h, 4 h, and 8 h after surgery, we found that lactic acid values in both groups peaked at 4 h after surgery and then declined. The rate of lactic acid decline was significantly faster in the group with a higher sufentanil dosage than in the lower group. The difference was statistically significant (*P* < 0.05)

The patients were further divided into two groups with the mean sufentanil infusion rate (1.88 µg kg^−1^ h) as the cutoff point. Fifty-one pairs of patients were screened by propensity score matching analysis (PSM) (Table 3). The repeated measures ANOVA further identified that there was a significant difference in lactic acid levels at four time points(0.1 h, 2 h, 4 h and 8 h after surgery) in both groups (*P* < 0.001). At the same time, there were also differences between the two groups with different infusion rates of sufentanil (*P* = 0.03) (Fig. 1).

**Table 3:** Propensity score matching analysis of different infusion rates of sufentanil

Variable	Sufentanil infusion rates < 1.88 µg. kg-1. h-1 group(n = 51)	Sufentanil infusion rates > 1.88 µg. kg-1. h-1 group(n = 51)	*P* value
Female sex	25 (49.1)	16 (31.3)	0.43
Age (years)	54.29 ± 8.77	55.29 ± 13.75	0.66
Body surface area (m^2^)	1.71 ± 0.21	1.73 ± 0.16	0.47
Preoperative diabetes mellitus	7(13.7)	3 (5.8)	0.96
Preoperative atrial fibrillation	14 (27.4)	8 (15.6)	0.64
Preoperative hypertension	17 (33.3)	10 (19.6)	0.25
Preoperative EF value (%)	61 (58, 65)	62 (56, 68)	0.31
Preoperative left ventricle (end-diastolic diameter, cm)	51 (48, 54)	52 (48, 55)	0.09
Cardiopulmonary bypass time (min)	125.29 ± 49.47	122.27 ± 60.64	0.78
Aortic occlusion time (min)	94.39 ± 42.46	91.56 ± 51.73	0.76
Total operation time (min)	260 (210, 300)	225 (180, 300)	0.22
Total sufentanil dosage (ug)	420.58 ± 120.24	590.19 ± 146.29	< 0.001
Relative sufentanil infusion rate (µg kg^−1^ h^−1^)	1.41 ± 0.26	2.44 ± 0.44	< 0.001
8 h mean blood glucose level (mmol/L)	10.5 (8.6,12.1)	9.8 (8.6,11.2)	0.4
Infusion rate of propofol (mg/kg h)	6.9 (6.5,7.5)	7 (6.5,8)	0.45
8 h Total dopamine dosage (mg)	165.6 (115.2,196.8)	158.4 (141.6, 175.2)	0.4
8 h Total Epinephrine dosage (mg)	9 (17.6)	13 (25.4)	0.36

## Discussion

Lactic acidosis is often associated with poor clinical outcomes. Patients with lactic acidosis due to sepsis or hypoperfusion had a three-fold increase in mortality [12]. Lactic acid values were positively correlated with poor clinical prognosis [13]. Postoperative mortality was increased in patients with both intraoperative and postoperative hyperlactatemia [14–16]. In contrast, some other studies have taken a different view. One study has suggested that in patients undergoing mitral valve surgery, hyperlactatemia is common, but mortality is low [3]. Another study suggested that lactic acidosis in heart transplant patients can be alleviated rapidly without any treatment, and the overall prognosis is good [17]. Therefore, the mechanism of lactic acid elevation may be more meaningful than the lactic acid value in patient management. There may be other causes of lactic acidosis in addition to the common hypoxia of tissues.

There are currently recognized causes of lactic acidosis after heart surgery, such as prolonged cardiopulmonary bypass and aortic occlusion, poor preoperative heart function and epinephrine use [18]. In our study, logistic multivariate stepwise regression analysis revealed that EF, aortic occlusion time and sufentanil relative infusion rate were risk factors for lactic acidosis. Cardiopulmonary bypass has been known to be a risk factor for lactic acidosis for a long time. Cardiopulmonary bypass may cause lactic acidosis due to low tissue perfusion. The transition from pulsatile to nonpulsatile blood flow to the kidneys and liver is also an important reason. These two organs are the main organs for lactic acid clearance [19]. However, some recent studies have shown that this factor does not significantly contribute to postoperative hyperlactatemia in most patients after heart surgery [20, 21]. Low EF often indicates poor preoperative cardiac function and poor tissue perfusion. It causes hyperlactatemia, which can be explained by tissue hypoxia (type A). However, in addition to these same risk factors as in previous studies, we found, surprisingly, that the intraoperative sufentanil infusion rate was also an independent risk factor for lactic acidosis. At present, the relationship between intraoperative opioid (sufentanil) dosage and postoperative lactic acidosis has been poorly investigated.

To our knowledge, because of the wide recommended range of opioid doses, the amount of sufentanil used by different heart centers varies greatly. Therefore, we believe that the reason for lactic acidosis in some patients is that the amount of sufentanil is insufficient and the stress response in the body is not well suppressed, which causes the release of endogenous epinephrine. Currently, some studies have confirmed that epinephrine use is associated with postoperative hyperlactatemia and hyperglycemia [22–24]. This mechanism is called epinephrine-induced aerobic glycolysis. Epinephrine excites the β2 receptor, leading to an increase in glycogenolysis, fatty decomposition, and gluconeogenesis in the body [25, 26]. This can lead to an increase in plasma glucose, which is an independent risk factor for lactate acidosis [27]. Hyperglycemia may enhance the potential role of epinephrine in the pathogenesis of hyperlactic acid [10]. At the same time, free fatty acids can inhibit pyruvate conversion to acetyl-CoA. This leads to an increase in lactic acid production. Our study showed that there was no relationship between hyperglycemia and lactic acidosis, possibly because we routinely used insulin to control blood glucose after surgery.

Epinephrine stimulation of the B2 receptor can directly increase glycolysis or enhance the activity of Na+ -K+-ATPase to increase glycolysis. Hyperlactatemia can be caused by any disease or stimulus that increases the level of adrenaline in the body [28]. Median sternotomy cardiac surgery is associated with greater trauma. Therefore, if the intraoperative opioid dose is insufficient, the stress response is not well inhibited, which may lead to increased epinephrine secretion and further lead to hyperglycemia. In addition, epinephrine can increase glycolysis and glycogenolysis in skeletal muscle, increase lipolysis and decrease muscular proteolysis. This may be another reason for hyperlactatemia. Our study showed that exogenous epinephrine use was also not associated with lactic acidosis. This may be due to the low number of cases of epinephrine use (20.9%). However, epinephrine-induced hyperlactatemia may be the best explanation for the relationship between sufentanil and postoperative lactic acidosis.

Another feature of this type of acidosis is that the prognosis is not as bad as that of anoxic acidosis. Our observations suggest that the incidence of lactic acidosis after cardiac surgery is 17.6%. The majority of patients (62.5%) had elevated blood lactate > 2 mmol/L on ICU admission. However, within 24 h, 95.3% of all patients had normal lactate values. The mortality is low (0.9%). Hemodynamics were stable in most patients, and no special intervention was required.

One study showed that infusion of epinephrine resulted in a significant increase in lactic acid levels but no significant change in the L/P (lactate/pyruvate) ratio and no significant decrease in tissue ATP [29]. Many tissues produce pyruvate and lactic acid under good oxygen supply, known as aerobic glycolysis [30]. The increase in lactic acid caused by aerobic glycolysis is often transient, with lactic acid levels dropping to normal after approximately 12 h [31]. This is completely different from acidosis caused by hypoxia. Therefore, when a patient is found to have lactic acidosis in the ICU, the cause should be carefully investigated.

Furthermore, repeated measures ANOVA suggested that there was a difference in lactic acid between the two groups of patients with different infusion rates of sufentanil, further confirming the association between sufentanil dosage and lactic acidosis. The peak lactic acid level occurred 4 h after the operation in the two groups and then declined. The rate of lactic acid decline was significantly faster in the group with a higher sufentanil dosage than in the lower group. At the same time, lactic acid levels at four time points (0.1 h, 2 h, 4 h and 8 h after surgery) were also different. This suggests that the causes of lactate elevation after cardiac surgery are complex. Therefore, it is important to distinguish between patients with transient hyperlactatemia and those with persistent hypoxia in postoperative patient management. Our results provide an interesting factor.

### Limitations

Our study has several limitations. First, it is a retrospective study, and bias cannot be eliminated. Second, the study was conducted in only one heart center. Studies in different regions may have different findings because different regions may have different methods of management. However, it should be noted that there has been no prospective study on the relationship between lactate acidosis and opioids (sufentanil). A prospective study should be conducted in the future to refute or confirm our findings. Finally, although we analyzed the relationship between sufentanil and lactic acidosis with adjustment for age, sex, EF, aortic occlusion time and cardiopulmonary bypass time, there may be other confounders that influenced the association.

## Conclusions

The inadequate intraoperative infusion rate of sufentanil is an independent risk factor for lactic acidosis in patients who underwent valvular heart surgery involving cardiopulmonary bypass in addition to the heart ejection fraction and aortic occlusion time. We believe that the mechanism is stress-induced aerobic glycolysis, just like epinephrine-induced aerobic glycolysis. It is an interesting cause of lactic acidosis, which is helpful for postoperative patient management. At present, there are few studies on the relationship between opioids and lactic acidosis. Further studies are needed to determine the appropriate dosage of opioids during cardiac surgery.

## Data Availability

The datasets used and/or analysed during the current study are available from the corresponding author on reasonable request.
